# Integrative Modeling and Experimental Insights into 3D and 4D Printing Technologies

**DOI:** 10.3390/polym16192686

**Published:** 2024-09-24

**Authors:** Angel Cabrera Pereira, Vasudev Vivekanand Nayak, Paulo G. Coelho, Lukasz Witek

**Affiliations:** 1 Department of Biomedical Engineering, City College of New York, New York, NY 10031, USA; acabrer016@citymail.cuny.edu; 2 Department of Biochemistry and Molecular Biology, University of Miami Miller School of Medicine, Miami, FL 33136, USA; 3 Division of Plastic Surgery, DeWitt Daughtry Family Department of Surgery, University of Miami Miller School of Medicine, Miami, FL 33136, USA; 4 Biomaterials Division, NYU Dentistry, New York, NY 10010, USA; 5 Hansjörg Wyss Department of Plastic Surgery, NYU Grossman School of Medicine, New York, NY 10016, USA; 6 Department of Biomedical Engineering, NYU Tandon School of Engineering, Brooklyn, NY 11201, USA

**Keywords:** 3D printing, 4D printing, polymer science, fused deposition modeling, biodegradable polymers, smart materials, additive manufacturing, shape-memory polymers, hydrogels, sustainable manufacturing

## Abstract

This review focuses on advancements in polymer science as it relates to three-dimensional (3D) and four-dimensional (4D) printing technologies, with a specific emphasis on applications in the biomedical field. While acknowledging the breadth of 3D and 4D printing applications, this paper concentrates on the use of polymers in creating biomedical devices and the challenges associated with their implementation. It explores integrative modeling and experimental insights driving innovations in these fields, focusing on sustainable manufacturing with biodegradable polymers, a comparative analysis of 3D and 4D printing techniques, and applications in biomedical devices. Additionally, the review examines the materials used in both 3D and 4D printing, offering a detailed comparison of their properties and applications. By highlighting the transformative potential of these technologies in various industrial and medical applications, the paper underscores the importance of continued research and development. The scope of this review also includes an overview of future research directions to address current challenges, enhance material capabilities, and explore practical applications.

## 1. Introduction

Three-dimensional (3D) printing, also known as additive manufacturing, has revolutionized the fabrication of structures with complex geometries and customized parts from digital models [1,2]. This technology has found applications in various fields, including aerospace, automotive, and biomedical engineering [3,4]. In recent years, the capabilities of additive manufacturing have been further expanded by the emergence of four-dimensional (4D) printing, which introduces programmable morphological transformation, allowing 3D-printed structures to change their shape or properties in response to external stimuli [5,6,7,8]. Polymers play a crucial role in the development of 3D and 4D printing due to their versatility, processability, and unique physicochemical and biological properties [9,10]. The ability to further tailor these properties has enabled the fabrication of functional parts for various applications, such as biomedical devices, flexible electronics, and smart materials [11,12,13]. In 4D printing, polymers are especially significant as they can create dynamic and responsive structures that adapt to their environment [14,15,16,17].

Four-dimensional printing of polymers is particularly relevant in medicine due to its potential to create dynamic, responsive, and personalized medical devices. Polymers such as hydrogels and bioresorbable materials are often biocompatible, making them suitable for use in implants, tissue engineering scaffolds, and drug delivery systems without causing adverse tissue reactions [12,18,19]. Moreover, 4D-printed polymers can be designed to change their shape or properties in response to temperature, pH, or moisture, to name a few stimuli [7,17,20]. This ability to undergo physical and mechanical changes over time can be leveraged to create medical devices such as self-expanding stents or shape-memory sutures. Additionally, 4D printing enables the production of customized implants, prosthetics, and orthotics that provide better fit and improved functionality relative to traditional one-size-fits-all solutions. The compact and deployable design of 4D-printed devices also facilitates minimally invasive surgical procedures [12,18]. For instance, a 4D-printed device can be inserted through a small incision and subsequently expand or change shape in situ, reducing the need for large, open surgeries, thereby expediting patient recovery. By producing personalized and adaptable medical devices, 4D printing of polymers can help reduce the need for multiple surgeries and device replacements, leading to lower healthcare costs and improved patient outcomes [7,20]. Furthermore, polymer 4D printing can create smart drug delivery systems that release medications in a controlled and targeted manner [3,19]. Additionally, 4D-printed scaffolds can guide the growth and differentiation of cells, promoting tissue regeneration and repair [15,17].

Advancements in polymer science have driven significant innovations in 3D and 4D printing technologies. This review explores the integrative modeling approaches that enhance our understanding of material behavior during the printing process and the experimental insights that drive the development of new polymeric materials with tailored physicochemical and mechanical properties. By examining the interplay between modeling and experimentation, this paper aims to elucidate the current state and prospects of 3D and 4D printing in advancing polymer science and provide a deeper understanding of how these advancements can be leveraged to develop future applications in medicine, tissue engineering, and sustainable manufacturing. This review specifically targets the developments in 3D and 4D printing technologies that are relevant to biomedical applications, focusing primarily on polymer-based materials. The discussion is centered on how these technologies can be leveraged to create advanced medical devices, leaving a comprehensive review of other applications, such as electronics, high-temperature applications, and loaded structures, to future studies.

## 2. Current State of 3D Printing Techniques

### 2.1. Overview of 3D Printing Technologies

**Figure 1:**
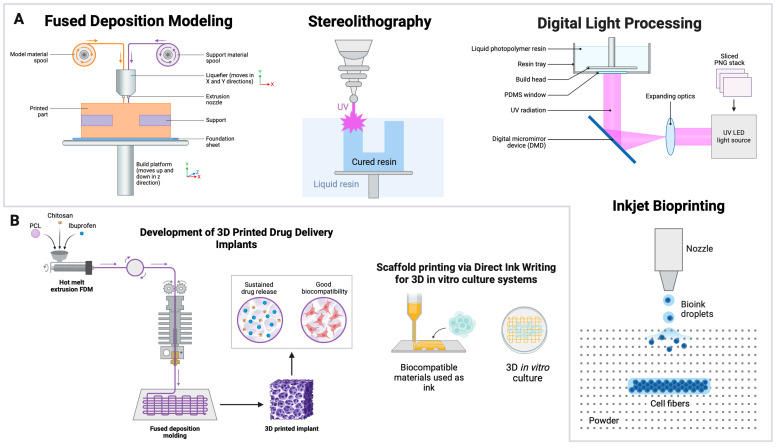
Schematic representation of some of the common 3D printing techniques and applications. (**A**) FDM extrudes thermoplastic filament through a heated nozzle, depositing material layer by layer. Stereolithography (SLA) process where a photopolymer resin is polymerized through the use of a UV source. Digital Light Polymerization (DLP) utilizes a digital projector screen to flash each layer’s image, thereby rapidly curing the photopolymer resin. Inkjet bioprinting provides a non-contact method of dispersing droplets containing bioinks and cells to create tissue-like structures for regenerative medicine. The figure encapsulates the diversity of methods used to achieve precise and complex 3D-printed structures. (**B**) Depiction of advanced applications, including the development of 3D-printed drug delivery implants via FDM and scaffold printing via Direct Inkjet Writing (DIW)—a type of inkjet printing for 3D in vitro culture systems. Created with BioRender.com.

Three-dimensional printing begins with a computer-aided design (CAD), where a digital model of the object is created. The model is then subsequently discretized and sliced, thereby generating a set of instructions for the 3D printer (e.g., gcode). The various 3D printing technologies available for use are categorized into several different types (Figure 1A), namely Material Extrusion, Vat Polymerization, Powder Bed Fusion, Material Jetting, Binder Jetting, Directed Energy Deposition, and Sheet Lamination. For instance, Material-Extrusion-based printers deposit the feedstock layer by layer, while other techniques, such as Vat Polymerization and Powder Bed Fusion, use a light or laser source to cure or fuse photosensitive polymer or metal powder feedstock. This sequential, layer-by-layer approach enables the creation of complex geometries and intricate details (Figure 1B) that are challenging to achieve with traditional, subtractive manufacturing methods. A comprehensive analysis of these techniques reveals the versatility and breadth of 3D printing technology, highlighting its potential across various fields.

#### 2.1.1. Material Extrusion

Fused Deposition Modeling (FDM), a type of Material-Extrusion-based 3D printing technology, is one of the most widely utilized methods in biomedical applications due to its cost-effectiveness and relative ease of use [21]. FDM involves extruding thermoplastic filaments which are fed into a heated nozzle, melted, and subsequently deposited layer by layer onto a build platform, where they solidify and bond to the previously extruded layers in a bottom-up approach. Recent advancements in FDM have focused on improving the resolution and surface finish of printed objects [22,23]. Techniques such as multi-material printing have enabled the creation of complex, functional parts with varied mechanical properties within a single print job [24]. For example, FDM has been utilized to develop scaffolds that facilitate and support cell growth and tissue regeneration [25], and in the production of custom-fit prosthetics with a higher strength-to-weight ratio, enhancing durability and user comfort [26], and compartmentalized drug delivery systems tailored for personalized treatment of sleep disorders [27]. Despite its versatility, FDM is limited by its relatively lower resolution compared to other techniques, such as SLA, and the potential for anisotropic properties due to the layer-by-layer deposition process. Surface roughness and weak interlayer adhesion are additional challenges.

#### 2.1.2. Vat Polymerization

Vat Polymerization techniques, such as Stereolithography (SLA) and digital light processing (DLP), utilize light to cure liquid photopolymer resins layer by layer. SLA uses a UV laser to trace each layer, curing the resin with high precision, while DLP uses a digital projector to flash entire layers, enabling faster print speeds and offering higher resolutions and smoother surface finishes, relative to FDM counterparts [28]. These technologies are particularly valuable in medicine for creating intricate surgical models, surgical guides, and dental prosthetics owing to the wider range of materials available [29]. For example, Karakurt et al. utilized SLA 3D printing to create ascorbic acid-loaded hydrogels, demonstrating controlled release properties. The authors developed a novel biocompatible resin consisting of poly(ethylene glycol) dimethacrylate (PEGDMA) and riboflavin (vitamin B2) as the photoinitiator, with ascorbic acid incorporated for drug delivery. The hydrogels displayed significant promise for personalized medicine by allowing customized drug geometries and dosages tailored to individual patient needs. Controlled drug release was achieved by optimizing the 3D printing process for various tablet geometries, resulting in effective encapsulation and release of ascorbic acid, making it a promising tool for advanced drug delivery systems [30]. In another study, Xu et al. explored the use of SLA to fabricate innovative bladder devices for intravesical drug delivery. These devices were designed to offer prolonged and localized drug delivery and are crucial for treating severe bladder diseases like interstitial cystitis and bladder pain syndrome. The study utilized an elastic resin to create hollow and solid devices loaded with lidocaine hydrochloride. The hollow devices released lidocaine completely within 4 days, while the solid devices enabled sustained release for up to 14 days. The printed devices displayed good mechanical properties, including resistance to compressive and stretching forces and quick recovery to their original shape, as well as acceptable blood compatibility and minimal hemolytic activity [31]. This capability to produce custom-designed devices that can release medication over extended periods represents a significant advancement in medical treatments and patient care.

DLP rapidly cures photopolymer resins at high resolutions. Borello et al. developed a mechanically tunable acrylate resin for multi-material 3D printing using DLP-SLA printers. This resin exhibited a wide range of elastic moduli by altering monomer and crosslinker ratios, enabling the creation of complex, mechanically heterogeneous structures with high resolution and structural integrity [32]. This study highlighted the versatility of DLP in producing precise and mechanically diverse structures, such as complex microfluidic devices. In another study, Gonzalez et al. developed a custom-made photopolymer based on acrylate PDMS, which enabled the production of complex-shaped microfluidic chips with excellent optical features, high chemical stability, and good mechanical properties [33], making them suitable for a wide range of biological and chemical analyses. SLA and DLP offer advantages such as high resolution and smooth surface finishes, making them suitable for intricate medical devices. However, the cured parts can be brittle, and the range of materials is limited compared to FDM. Furthermore, post-processing steps, including washing and curing, are essential to achieve desired mechanical properties and biocompatibility.

#### 2.1.3. Powder Bed Fusion

Selective Laser Sintering (SLS) and Selective Laser Melting (SLM) are Powder Bed Fusion technologies that use a laser to sinter or melt powdered materials. SLS is commonly used for polymers and certain metals, producing strong, durable parts, while SLM is primarily used for metals, fully melting the powder to form solid objects [34,35]. While SLS can produce parts without support structures, as the powder bed itself provides support, SLM requires supports for thermal management during metal printing to prevent warping. These technologies are used in creating customized implants with excellent mechanical properties and biocompatibility, such as poly(L-lactic acid) (PLLA) scaffolds loaded with dexamethasone for bone regeneration. Sun et al. demonstrated the use of SLS to fabricate PLLA porous scaffolds loaded with dexamethasone for bone regeneration. The scaffolds maintained their mechanical integrity and supported controlled drug release, showcasing the potential of SLS in creating customized medical implants with excellent mechanical properties and biocompatibility [36].

Hu et al. investigated the improvement of corrosion resistance in AZ91D magnesium alloy, prepared via SLM and subjected to T4 heat treatment, for biomedical applications. The authors demonstrated that the T4 treatment dissolved β-Mg17Al12 phases, refined the microstructure, and enhanced corrosion resistance by reducing corrosion current density and hydrogen evolution rate [37]. The treated alloy exhibited better uniform corrosion and maintained acceptable mechanical properties, making it a promising candidate for biodegradable medical implants. Furthermore, a recent study explored the use of SLM to fabricate titanium-based models with controlled surface morphological features. The research aimed to assess the capability of SLM in producing surfaces with specific textures, such as dimples and bumps, which are important for biomedical implants. Various test samples were built using Ti_6_A_l4_V powder, and the surface morphology was analyzed based on the orientation during the SLM process. The results demonstrated that SLM effectively produced surface features with high precision, but the accuracy of these features varied depending on their orientation on the build platform. For instance, surfaces built at a 45° angle exhibited a significant “staircase” effect, while vertical and horizontal orientations showed better feature definition [38]. The study also highlighted the importance of process control in achieving consistent surface textures, with implications for the design of implants where surface porosity and texture are crucial for osseointegration and biological compatibility. SLS and SLM enable the production of complex geometries and high-strength components suitable for load-bearing applications. However, the high cost of materials and equipment, as well as the need for precise heat management in SLM, are significant drawbacks.

#### 2.1.4. Material Jetting

Material Jetting or PolyJet 3D printing is an advanced additive manufacturing technique that employs a layer-by-layer approach to jetting liquid photopolymers onto a build platform, curing each layer with UV light. The process is highly precise, allowing for layers as thin as 16 microns, enabling the creation of intricate geometries and smooth surfaces. One of PolyJet’s key advantages is its ability to blend multiple materials in a single print, making it possible to fabricate objects with varying mechanical properties, textures, and even colors. This flexibility is particularly useful in fields like medical device manufacturing, where materials with different hardness or flexibility are required to mimic the properties of human tissues. Additionally, PolyJet’s use of soluble support materials allows for the creation of highly complex internal structures without the need for laborious post-processing.

Several studies highlight the practical applications and performance of PolyJet printing in medical and industrial contexts. Bochnia et al. investigated the mechanical and rheological behavior of MED610, a biocompatible resin used in medical models, including full and hexagonal cellular structures. Their research demonstrated that using filled cellular structures significantly improves tensile strength and flexibility [39], critical for applications like prosthetics and surgical models, where lightweight yet durable structures are needed. Similarly, Emiliani et al. focused on the post-processing effects on photopolymer resins such as Agilus30, showing that aging and glycerol treatment can impact material flexibility [40], which is vital for surgical simulators that must retain their mechanical properties over time. Turek et al. provided a comprehensive evaluation of the dimensional accuracy and surface roughness of models created with PolyJet technology, revealing that materials like Digital ABS Plus and Vero Clear offer high geometric precision [41], making PolyJet particularly suited for industries requiring tight tolerances and smooth finishes. Together, these studies underscore PolyJet’s transformative role in producing complex, functional prototypes and models across medical and industrial sectors, combining precision with material versatility. The collective analysis shows that PolyJet technology not only supports diverse material combinations but also delivers high-performance outputs with minimal post-processing, positioning it as a critical tool in modern manufacturing.

#### 2.1.5. Bioprinting and Direct Ink Writing (DIW)

Beyond the traditional 3D printing techniques, advanced bioprinting methods are gaining prominence for their ability to precisely manipulate biological materials. Inkjet bioprinting is a non-contact technique that disperses droplets containing cells and bioinks, including continuous inkjet and drop-on-demand methods [25]. This technique is employed to create tissue-like structures for regenerative medicine, offering high precision in cell placement [25]. In the pharmaceutical industry, inkjet bioprinting has been used to develop personalized drug delivery systems, such as polypills, which improve patient compliance and treatment outcomes [24]. Laser-Induced Forward Transfer (LIFT) uses a laser to transfer cells or materials onto a substrate with micrometer-level precision, avoiding nozzle clogging issues [34]. LIFT is utilized for creating cellular constructs with precise cell placement, essential for tissue engineering applications [34].

A variation of an inkjet-based technique, Direct Ink Writing (DIW), involves the extrusion of highly viscous materials through a nozzle, enabling the controlled deposition of various biomaterials such as ceramics and hydrogels. This has proven effective in creating bone scaffolds with high structural integrity and mechanical properties that support bone regeneration [42,43,44,45,46,47,48,49,50,51]. For instance, Bhardwaj et al. demonstrated the development of advanced hydrogel inks specifically designed for DIW that possess the necessary rheological properties for stable extrusion and rapid modulus recovery, making them ideal for biomedical applications [52]. Similarly, others explored the use of cryo-assisted DIW to fabricate organohydrogels with enhanced impact resistance, integrating intrinsic and configural superiority to achieve superior mechanical performance [53]. The versatility of DIW extends to the production of polymer composite structures. The integration of β-tricalcium phosphate (β-TCP) with polymers like collagen, as demonstrated by Cabrera Pereira et al., enhanced cell viability and proliferation, making these scaffolds suitable for bone tissue engineering applications [54]. Furthermore, Pacheco-Vergara et al. compared 3D-printed mesoporous bioactive glass (MBG), bioglass 45S5, and β-TCP scaffolds, demonstrating superior cell viability and osteogenic differentiation in β-TCP scaffolds [55], which is essential for effective bone regeneration. Lastly, the combination of polylactic acid (PLA) with β-TCP in 3D-printed scaffolds provided promising results for bone tissue regeneration. The rheological properties, cellular viability, and thermomechanical behavior of these composites indicate their potential for customized, patient-specific applications in bone tissue engineering [56].

Three-dimensional printing technologies offer diverse capabilities for producing complex and functional parts, particularly in the biomedical field. Each technology has unique advantages and limitations, influencing its suitability for different applications. FDM, SLA, and SLS each offer distinct benefits in terms of material use, resolution, and part strength, while advanced techniques like DIW and inkjet bioprinting provide precision in biomedical applications. Understanding these differences is crucial for selecting the appropriate method for specific biomedical needs. Future advancements in material science and printing techniques will further expand the possibilities of these technologies, driving innovation in personalized medicine and sustainable manufacturing. Table 1 summarizes the various 3D printing methods utilized in biomedical applications discussed in this review. For more extensive reviews on 3D printing technologies for various applications, we recommend [57,58].

### 2.2. Fused Deposition Modeling as a Cornerstone in 3D Printing

FDM has become a transformative technology in the biomedical field, revolutionizing how medical devices, prosthetics, implants, and other biomedical applications are designed and manufactured. The integration of advanced composite materials has significantly enhanced the mechanical and functional properties of 3D-printed parts, while the optimization of printing parameters has led to superior performance. These advancements are not only improving the quality and customization of biomedical devices but are also pushing the boundaries of what is possible in personalized medicine and tissue engineering.

#### 2.2.1. Material Innovation

**Figure 2:**
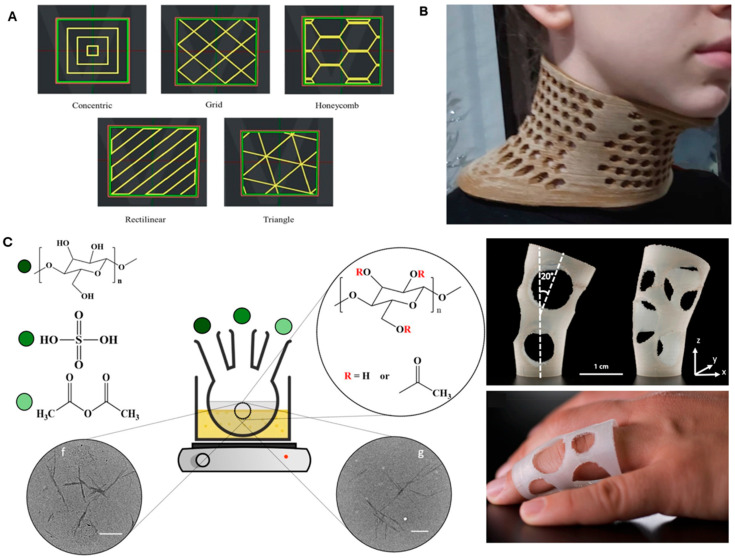
Innovations in biodegradable composites, thermoplastic materials, and PLA/copper composites for enhanced mechanical properties and biomedical applications. (**A**) PLA/copper composites with varying infill patterns significantly enhance mechanical properties (reproduced with permission from [63]; *Materials Today: Proceedings*, Elsevier, 2023). (**B**) New thermoplastic materials composed of PLA and organic bio-plastic compounds demonstrate applications such as custom neck orthoses, offering reduced production costs and environmental impact (reprinted from [69], under the terms of the Creative Commons License CC BY 4.0—https://creativecommons.org/licenses/by/4.0/ (accessed on 31 July 2024). (**C**) Giubilini et al. developed biodegradable composites using PHBH reinforced with CNCs via a solvent-free melt-compounding process, with an application in flexible medical supports shown; scale bars = 150 nm. Reprinted from [70] under the terms of the Creative Commons License CC BY 4.0—https://creativecommons.org/licenses/by/4.0/ (accessed on 31 July 2024).

Material innovation has been a focal point in FDM research. Amin et al. demonstrated the use of PLA and hydroxyapatite composites to create biocompatible scaffolds with improved mechanical properties, suitable for bone tissue engineering [61]. Similarly, Hlaváčiková et al. explored the customization capabilities of FDM, creating patient-specific implants that showcase the technology’s potential in personalized medicine [62]. Kumaresan et al. investigated PLA and copper composites, finding that varying infill patterns (Figure 2A) and compositions can significantly enhance mechanical properties [63], making these materials suitable for application in electronics. Research into optimizing composite formulations, such as PLA mixed with natural fillers, also aims to improve environmental sustainability and mechanical properties [64].

The range of materials used in FDM is broad, encompassing various thermoplastic polymers such as polylactic acid (PLA), polyamide (PA), acrylonitrile butadiene styrene (ABS), and polyethylene terephthalate glycol (PETG). High-performance materials like polyether ether ketone (PEEK) and polyetherimide (ULTEM) are employed for applications demanding superior mechanical and thermal properties [65,66]. Fiber-reinforced filaments incorporating carbon, glass, or Kevlar fibers also enhance the mechanical properties of the printed constructs [63,67]. Innovations such as polymer blends of PLA with polybutylene adipate-co-terephthalate (PBAT) and bioresorbable-peptide-incorporated polymers are also being explored to improve functionality and application scope [21,68].

Recent research has introduced five new thermoplastic materials composed of PLA and organic bio-plastic compounds [69]. These materials are made using agricultural byproducts. They offer advantages such as reduced production costs and environmental impact while maintaining or enhancing mechanical properties. Applications in biomedical fields, such as custom neck orthoses (Figure 2B) and laryngoscopes, demonstrate their potential. Giubilini et al. developed biodegradable composites using poly(3-hydroxybutyrate-co-3-hydroxyhexanoate) (PHBH) reinforced with acetylated cellulose nanocrystals (CNCs) via a solvent-free melt-compounding process (Figure 2C). This method significantly improved the mechanical and thermal properties of PHBH, increasing the storage modulus by 150% and the thermal stability to 265 °C. The composites were successfully 3D-printed into eco-friendly materials, demonstrating enhanced biodegradability with a 94% weight loss after 78 days under composting conditions [70].

#### 2.2.2. Printing Parameter Optimization and Mechanical Properties

**Figure 3:**
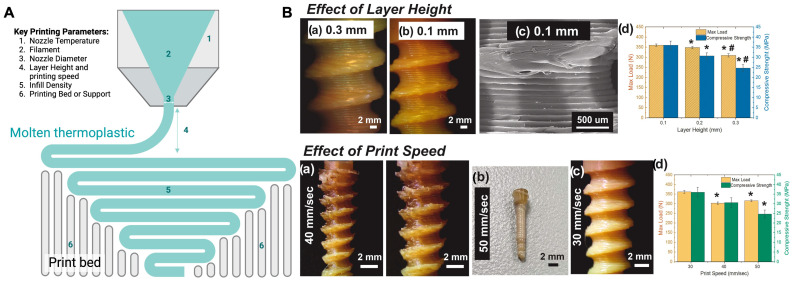
Key printing parameters and their effects on FDM 3D-printed part quality. (**A**) A schematic representation of key FDM printing parameters, including nozzle temperature, filament type, nozzle diameter, layer height, infill density, and bed temperature, that influence the quality of the final print. These parameters are critical for determining the overall performance of FDM-printed parts, including surface finish, structural integrity, and material properties. Created with BioRender.com. (**B**) The effects of varying layer height and print speed on surface roughness and mechanical properties. The comparison highlights how adjustments in these parameters can improve print resolution, interlayer adhesion, and mechanical strength. Additionally, compressive strength results for varying layer heights and print speeds (d) demonstrate the trade-offs between resolution and mechanical performance in FDM-printed parts; * means *p* < 0.05; # means statistically significant with respect to the maximum load or compressive strength. Reprinted from [66] under the terms of the Creative Commons License CC BY 4.0—https://creativecommons.org/licenses/by/4.0/ (accessed on 9 September 2024).

Optimizing printing parameters is crucial for improving the quality and performance of FDM-printed parts. Key parameters include layer height, printing speed, nozzle temperature, bed temperature, and infill density (Figure 3A). Research has shown that nozzle geometry, such as square-shaped nozzles, can significantly enhance surface finish by reducing roughness [23]. Sonaye et al. optimized the printing parameters of 3D-printed PEEK-based dental implants (Figure 3B), which exhibited excellent mechanical properties [66]. They identified optimal printing parameters, including a print speed of 30 mm/s, a layer height of 0.1 mm, a nozzle temperature of 450 °C, a bedplate temperature of 150 °C, and a chamber temperature of 90 °C. These parameters enhanced the print resolution, interlayer adhesion, and crystallinity of the PEEK implants, resulting in superior compressive strength and fatigue resistance, even after simulated aging equivalent to 30 years of in vivo performance [66]. Studies have also focused on the impact of printing speed on tensile strength, with slower speeds generally resulting in higher tensile strength but also higher residual stresses [71]. In situ thermal treatments during printing have been employed to improve interlayer adhesion, as demonstrated with polyetherimide (PEI) parts using a radiant heating system set at 390 °C and a printing speed of 35 mm/s, resulting in a 183% increase in tensile strength, a 22% increase in elastic modulus, and a 190% increase in elongation at break due to enhanced layer bonding and reduced porosity [65].

The mechanical properties of FDM-printed parts are influenced by the printing parameters and the inherent characteristics of the materials used. For instance, the inclusion of copper in PLA filaments can significantly increase tensile strength and stiffness, making these composites suitable for high-performance applications [63]. However, challenges such as weak interlayer adhesion, surface roughness, and thermal stresses persist. Research into layer thickness variations has shown that lower thicknesses generally improve impact properties, but the trade-offs in printing time and material usage must be considered [22]. Moreover, achieving consistent mechanical properties across different geometries and printing conditions remains a complex task, necessitating robust numerical models to predict and optimize outcomes [72].

#### 2.2.3. Applications

FDM technology is widely used across various industries due to its ability to produce custom, on-demand parts. For instance, biocompatible polymers like PLA and PA have been deposited on titanium alloys to improve the functionality of endoprostheses [73]. Additionally, the mechanical properties of natural-fiber-reinforced polymers showcase their potential for sustainable and high-performance biomedical devices [64]. FDM’s use in creating complex anatomical models is invaluable for surgical planning and medical education, especially with the combination with CAD to enhance the precision and functionality of printed medical devices [74,75]. Šibalić et al. focused on optimizing the FDM process for PVA, highlighting its potential for medical applications due to its biocompatibility and water solubility [76].

Recent innovations in FDM have significantly advanced biomedical applications. The development of 3D-printed vaginal drug delivery systems by using thermoplastic polyurethane (TPU) filaments is crucial for treating bacterial vaginosis, where the flexibility of TPU allowed for the creation of vaginal rings that are manually filled with jellified metronidazole or chloramphenicol [77]. These filament rings demonstrated suitable dissolution profiles and biocompatibility, making them a promising alternative for localized treatment, thereby improving patient compliance and treatment effectiveness. In addition, bi-compartmental tablets for controlled drug release also demonstrate the technology’s potential in pharmaceutical applications [78], showing promise in enhancing drug delivery systems, particularly for protecting thermolabile drugs.

In bone tissue engineering, the fabrication of scaffolds from poly(3-hydroxybutyrate) (PHB) and polycaprolactone (PCL) blends, reinforced with tricalcium phosphate (TCP), has shown great potential. These scaffolds, processed via FDM, exhibit enhanced mechanical properties comparable to human trabecular bone and support cell proliferation and differentiation [79]. The addition of TCP significantly improves the osteoconductive properties of the scaffolds, making them suitable for bone regeneration applications. Multifunctional polymer nanocomposite scaffolds made from drug-loaded halloysite nanotube (HNT)-reinforced polylactic acid (PLA) have also been developed using FDM [80]. These scaffolds have exhibited improved tensile, compression, and flexural strengths, making them robust for various biomedical applications [80]. The incorporation of metformin-loaded HNTs has allowed for controlled drug release, enhancing the scaffold’s functionality in bone regeneration and other tissue engineering applications.

Beyond the scope of biomedical applications, 3D printing technologies have seen significant advancements in other sectors. Conductive materials for electronics, composite materials for loaded structures, and ceramic-based materials for high-temperature applications represent key areas of innovation. For instance, conductive polymers such as polyaniline and polypyrrole have been explored for use in 3D-printed electronic components, enabling flexible and lightweight designs. In structural applications, fiber-reinforced composites are gaining traction due to their superior strength-to-weight ratios. Ceramic materials, capable of withstanding extreme temperatures, are being developed for the aerospace and automotive industries. These advances highlight the versatility of 3D printing technologies, although a detailed exploration of these fields is beyond the scope of this review.

Future research directions involve improving the scalability of FDM for mass production and integrating smart materials that can respond to environmental stimuli. Comparative studies with other 3D printing methods, such as Stereolithography (SLA) and Selective Laser Sintering (SLS), highlight FDM’s unique benefits and limitations, guiding further innovations in this field [72]. The ongoing advancements in material science and printing technology continue to expand the capabilities and applications of FDM, making it a cornerstone of modern additive manufacturing.

### 2.3. Implications of 3D Printing for Polymer Science

Advancements in 3D printing, particularly within polymer-based technologies such as FDM, have significant implications for the field of polymer science. The integration of sophisticated 3D printing techniques with novel polymer materials is driving innovation and expanding the possibilities in biomedical applications. These developments are enabling the creation of polymers with customized properties, such as enhanced mechanical strength, biocompatibility, and biodegradability. This necessitates a deep understanding of polymer chemistry and material science to tailor the properties of these materials for optimal performance in 3D printing processes.

A key implication is the ability to design and produce polymers with specific mechanical properties. For instance, fiber-reinforced polymers incorporating materials like carbon, glass, or Kevlar fibers offer improved tensile strength, stiffness, and thermal stability. These attributes make such materials suitable for demanding applications such as bone scaffolds and medical implants. Biocompatibility is another critical area where 3D printing is making strides. The use of biocompatible polymers like PLA, PEEK, and bioresorbable materials ensures that implants and scaffolds can be safely integrated into the human body without causing adverse reactions. Additionally, incorporating bioactive compounds, such as β-tricalcium phosphate (β-TCP), into these polymers further enhances their ability to promote cell viability and tissue regeneration.

The development of smart polymers that respond to environmental stimuli such as temperature, pH, and light is another exciting frontier in polymer science. These materials can be integrated into 3D printing processes to create adaptive biomedical devices that can change their properties in response to physiological conditions. Such advancements could lead to the creation of implants that release drugs on demand or scaffolds that dynamically support tissue growth, thus improving the efficacy of medical treatments.

Sustainability is also becoming a focal point in polymer science. Exploring eco-friendly polymers and composites, such as those derived from renewable sources or incorporating natural fillers, is essential for reducing the environmental impact of 3D printing [64]. Innovations in biodegradable and bioresorbable polymers also align with the growing emphasis on sustainable biomedical solutions, as these materials can reduce the need for secondary surgeries to remove implants.

Looking ahead, the scalability of 3D printing processes will need enhancement to enable mass production of polymer-based medical devices while maintaining quality and precision. Moreover, developing advanced materials that support cell viability and promote specific cellular functions and tissue regeneration will be essential. Combining 3D printing with other advanced manufacturing technologies, such as 4D printing, microfluidics, and nanotechnology, promises the creation of multifunctional biomedical devices. The expansion of customized polymer-based implants and devices tailored to individual patient needs, leveraging advancements in CAD and printing technologies, will further personalize medical treatment [74]. The synergy between polymer science and 3D printing is driving transformative advancements in biomedical applications. Continuous innovation in polymer materials, combined with the precision and versatility of 3D printing technologies, is opening new frontiers in medical treatment and patient care, ultimately improving health outcomes and quality of life.

## 3. The Emerging Realm of 4D Printing

In recent years, 4D printing has emerged as an exciting advancement in additive manufacturing, introducing a new dimension—time—to static 3D-printed structures. First introduced in 2013 by Skylar Tibbits, 4D printing combines stationary 3D-printed constructs with time-responsive behavior, enabled by stimuli-responsive materials such as shape-memory polymers (SMPs), hydrogels, and composite materials [8]. These materials allow printed structures to autonomously transform their shape, function, or properties over time in response to stimuli such as heat, light, humidity, or pH, to name a few. This shift from static to dynamic structures represents a significant leap forward, making 4D printing highly relevant for applications requiring adaptability and multifunctionality, such as biomedical implants, self-healing materials, and soft robotics. While several reviews have explored the principles and applications of 3D printing, there is a growing need to understand the transition to 4D printing, particularly in the context of its novel materials and multifunctional applications. This review aims to highlight recent breakthroughs in 4D printing, focusing on key innovations in smart materials, material–process–property correlations, and transformative applications across the biomedical industry.

**Figure 4:**
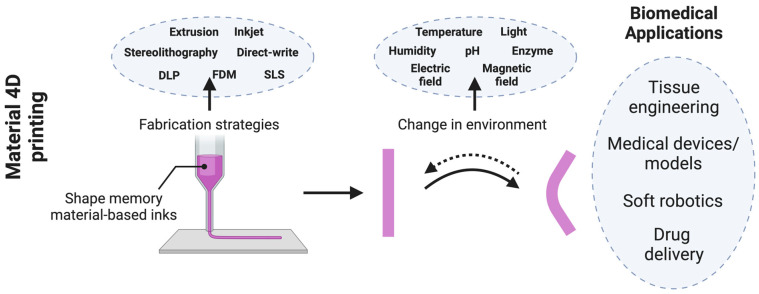
Overview of 4D printing in biomedical engineering. Created with BioRender.com.

Since its inception, 4D printing has garnered significant attention from both the public and the research community focused on smart materials and 3D printing technologies. While early definitions of 4D printing simply characterized it as the combination of 3D printing with time being the fourth dimension [81], the concept has evolved significantly. Today, 4D printing is widely understood as the ability of a 3D-printed object to alter not only its shape but also its properties and functionality over time in response to external stimuli (Figure 4). This section of the review delves into the advancements in 4D printing, emphasizing smart materials, material–process–property correlations, and its current and future applications.

### 3.1. Materials and Processes in 4D Printing

The functionality of 4D printing hinges on the integration of smart materials that can respond to external stimuli, enabling the dynamic, time-based transformations characteristic of this technology. This section explores the key materials and processes that underpin the evolution of 4D printing, with a focus on shape-memory polymers (SMPs), hydrogels, and composite materials.

#### Shape-Memory Polymers (SMPs)

**Figure 5:**
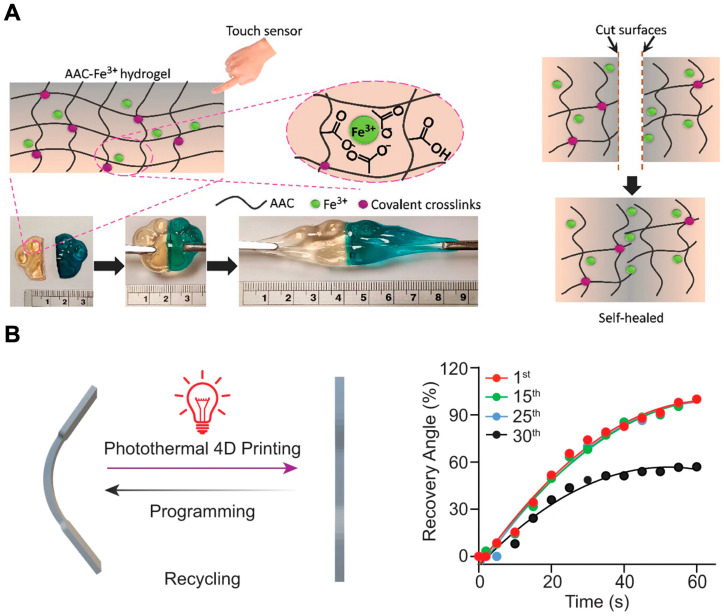
Shape-memory polymers (SMPs) in 4D printing. (**A**) Illustration of the structure and functionality of a dually crosslinked acrylic acid hydrogel reinforced with Fe^3+^ ions. The diagram shows how the hydrogel, once cut, can self-heal through the reformation of covalent crosslinks, making it suitable for stretchable electronics and soft robotics applications. (**B**) The photothermal actuation process of 4D-printed structures is demonstrated. These structures can be programmed and recycled, displaying dynamic shape recovery when exposed to near-infrared light. The graph shows its shape-memory performance and durability, highlighting the high consistency of the recovery angle over time and showcasing the material’s suitability for applications requiring robust mechanical and biocompatible properties, such as soft robotics. Reprinted from [83,84] under the terms of the Creative Commons License CC BY 4.0—https://creativecommons.org/licenses/by/4.0/ (accessed on 9 September 2024).

Shape-memory polymers are pivotal in 4D printing due to their ability to return to a pre-defined shape when exposed to stimuli such as heat, light, or magnetic fields. Some SMPs can even exhibit pH-responsive actuation, enabling precise shape memory programming [82]. The development of high-strength, high-toughness SMPs underscores their versatility in creating durable and adaptable structures. For instance, Li et al. introduced a dually crosslinked acrylic acid hydrogel reinforced with Fe^3+^ ions (Figure 5A), demonstrating exceptional properties with a gauge factor of 3.93 under a strain of 1500%, a fracture strain up to 1700%, and self-healing efficiencies of 88% for mechanical and 97% for electrical properties, making it suitable for stretchable electronics. The acrylic acid hydrogel’s 4D printability enabled the creation of shape-morphing and self-healing structures, with potential applications in sensors and soft robotics [83].

Similarly, the liquid metal–polymer composites described by Zhang et al. utilize near-infrared (NIR) light to induce shape changes (Figure 5B). These composites are created using a unique approach, where liquid metal nanoparticles (LMNPs), grafted with RAFT (Reversible Addition–Fragmentation Chain Transfer) agents, are integrated into UV-cured 3D-printed polymers. The RAFT agents improve the stability and dispersity of the nanoparticles within the polymer matrix. Under NIR light, these composites exhibit photothermal-responsive behavior, enabling them to restore pre-programmed shapes in under 60 s. LMPCs maintain shape recovery efficiency through multiple cycles of deformation and recovery, making them particularly suitable for soft robotics and biomedical devices due to their biocompatibility and mechanical properties, such as reduced glass transition temperature (T_g_) and enhanced flexibility. Additionally, LMPCs are capable of lifting objects up to five times their weight during shape recovery, demonstrating their potential for engineering artificial muscles [84].

In addition to these advancements, the integration of dynamic imine bonds in SMPs has enabled permanent shape reconfigurability by allowing topological rearrangements in the polymer network through the reversible exchange of imine bonds under relatively mild conditions, without the need for catalysts. This cutting-edge approach facilitates both temporary shape fixation and permanent shape alteration, providing a versatile platform for adaptive structures. For instance, Miao et al. demonstrated that their 4D-printed methacrylate systems, enhanced with a hyperbranched crosslinker (HPASi), exhibit excellent shape memory properties, with a shape fixity of 97.5% and shape recovery ratios of up to 93.7%. Additionally, these systems can undergo stress relaxation, allowing for plastic deformation and shape reconfiguration [85]. Furthermore, the incorporation of fractal-based stretchable circuits into SMPs localized heating and precise shape transformations, enhancing their functionality in complex medical applications [86].

SMPs that are thermoresponsive have also gained attention for their ability to undergo autonomous 2D-to-3D transformations upon heating, highlighting the importance of temperature as a stimulus in SMPs [87]. For example, triple-shape memory cyanate composites based on interpenetrating polymer networks demonstrate remarkable mechanical properties and shape recovery behaviors [88]. Similarly, highly stretchable elastomers have shown the ability to recover their original shapes after significant deformation and heating to 70 °C and the fixing of their temporary shape by cooling [89]. These elastomers were also capable of self-healing by heating to 80 °C, allowing the PCL chains to diffuse and re-entangle, effectively closing the cracks [89]. This process restored part of the mechanical strength of the semi-interpenetrating polymer network elastomer, including the ability to regain stretches of up to 600% [89].

Recent advancements in vat photopolymerization techniques have further expanded the potential of SMPs for creating smart structures with rapid and efficient shape transformations [90]. Alam et al. further demonstrated the potential of SMPs in creating smart structures with rapid and efficient shape transformations using vat photopolymerization. They created a photocurable SMP resin from a liquid crystal RM257 mixed with a commercial acrylic-based resin. When heated above the nematic–isotropic phase transition temperature, the material was rendered elastic and programmable into temporary shapes. Upon cooling, shapes were able to be retained until reheated, at which point constructs returned to their original form [91]. This study also used finite element analysis to demonstrate the tunability of the mechanical properties resulting from the programming steps.

Researchers have also continued to develop advanced materials, including composites with enhanced properties, which are crucial for expanding the application scope of 4D printing in both biomedical and engineering fields [92]. For example, the use of bioinspired dynamic spider silks with tunable mechanical properties showcases innovation in material development for 4D printing applications [93]. Abdullah et al. introduced 4D-printed hydrogels based on poly(acrylic acid) and hexadecyl acrylate, demonstrating reversible strong-to-weak gel transitions near body temperature and exceptional mechanical properties with a Young’s modulus of up to ~215 MPa and toughness of up to ~7 MJ/m^3^, providing a significant advancement for tissue engineering applications [94]. These authors demonstrated the successful integration of multiple smart materials into a single 4D-printed structure, allowing for multifunctional properties such as shape memory, self-healing, and conductivity [95]. Notably, the development of submicron 4D printing of SMPs enables the creation of complex, multi-color invisible inks with high-resolution features, expanding the potential applications in anti-counterfeiting and secure information storage [96].

### 3.2. Hydrogels and Composite Materials

Hydrogels and composite materials play a critical role in 4D printing, particularly in biomedical applications. Ren et al. introduced lignin-based hydrogel actuators that harness photothermal conversion for light-driven actuation [97], demonstrating the potential of composite hydrogels in creating responsive, multifunctional materials. Hydrogel-based actuators with diffusion-path architecture design demonstrate programmable deformation properties, suitable for minimally invasive medical implants [98]. In another example, swelling–stiffening hydrogel scaffolds have been developed to demonstrate similar programmable deformation properties, emphasizing their potential use in medical implants [99].

Further advancements in hydrogel technologies, such as the use of reactive-oxygen-species-responsive hydrogels for smart drug release systems, have paved the way for more targeted and controlled delivery mechanisms. This approach, as studied by Regato-Herbella et al., leverages the oxidative response in biological environments to trigger the release of therapeutics, providing a precise and responsive solution for medical treatments [100]. Additionally, Hiendlmeier et al. demonstrated the development of self-folding hydrogel-based electrodes, further highlighting the versatility of hydrogels in biomedical applications, where their self-folding nature enhances their utility in bioelectronic devices [101].

The latest progress also includes the development of soft microactuators capable of dynamic responses to changes in the surrounding medium, positioning them as viable options for targeted drug delivery and microsurgical applications [102]. Notably, Luo et al. have advanced biocompatible scaffold technology using in situ photo-crosslinking, highlighting the continued innovation in hydrogel technologies for medical uses [103]. Additionally, Pan et al. reviewed the development and applications of chitosan-based self-healing hydrogels, underscoring their potential in tissue regeneration, drug delivery, and biosensing, further solidifying the role of hydrogels in next-generation biomedical applications [104].

The versatility of SMPs and hydrogels is further illustrated by their applications across various industries. For instance, the combination of benzoxazine and epoxy thermosets for UV-assisted Direct Ink Writing produces materials with enhanced toughness and thermal stability, making them highly suitable for applications that demand durability and responsiveness [105]. Similarly, Weng et al. demonstrated how glass-fiber-regulated shape-shifting structures can be tailored to achieve specific mechanical properties and functionality, highlighting the importance of multi-material integration in composite development [106].

The integration of materials and stimuli-responsive behaviors is crucial for advancing the capabilities of 4D printing. For example, 4D-printed biodegradable shape-memory scaffolds, created using a four-axis 3D printing system, have demonstrated improved mechanical properties and shape recovery efficiencies, positioning them as ideal candidates for biomedical applications [107]. Furthermore, temperature-responsive poly (N-vinyl caprolactam) (PNVCL) hydrogels exhibit reversible expansion and shrinkage behavior, ideal for creating smart and adaptive structures [108]. Further work by Zolfagharian et al. on multi-material 4D printing with tunable bending models emphasizes the importance of understanding material interactions in achieving complex shape transformations [109]. The ability to tune the mechanical properties and responsiveness of printed materials through careful selection and combination of components opens new possibilities for creating smart and adaptive structures. In line with this, de Kergariou et al. further support the advancement of composite materials in achieving programmable shape transformations by creating flax-fiber-reinforced hydrogels [110]. The integration of advanced nanomaterials, such as high-performance triboelectric nanogenerators based on 2D materials, further expands the utility of 4D printing energy-harvesting biomedical applications [111]. The work on multidimensional crosslinked networks by Luo et al. further illustrates the importance of material innovation in achieving rapid, reconfigurable, and re-foldable structures [112]. Finally, the development of shape-memory vascular stents based on βCD-g-polycaprolactone further exemplifies the potential of 4D printing in creating advanced medical devices with superior mechanical and biocompatible properties [113].

### 3.3. Material–Process–Property Correlations and Applications in 4D Printing

The stimuli-responsive behavior of 4D-printed materials is integral to their functionality. Understanding the correlations between materials, processes, and properties is crucial for optimizing their performance. By tuning variables such as material composition and processing techniques, researchers achieve specific outcomes like improved shape retention, increased tensile strength, and enhanced actuation stability. This section delves into these correlations, focusing on shape-memory polymers, hydrogels, and other polymer composite materials, as well as their applications in biomedical and engineering fields.

#### 3.3.1. Shape-Memory Polymers (SMPs)

SMPs are among the most studied materials in 4D printing due to their unique ability to return to a pre-programmed shape when exposed to stimuli. Miao et al. investigated SMPs using soybean oil epoxidized acrylate (SOEA) to create biocompatible scaffolds suitable for biomedical applications. They employed Stereolithography to control the polymerization of SOEA, resulting in scaffolds that support human mesenchymal stem cells (hMSCs). Key variables included print speed and laser frequency, which affected the scaffold’s thickness, width, and surface structure. The scaffolds demonstrated excellent shape-memory properties, fixing a temporary shape at −18 °C and recovering fully at 37 °C [18]. Similarly, Alam et al. explored the potential of thermoresponsive SMPs by utilizing vat photopolymerization techniques, specifically digital light processing (DLP), to achieve rapid and efficient shape transformations. Their use of a customized resin combined with liquid crystals (LCs) enabled tunable mechanical properties in SMP-based structures, which were optimized for applications in soft robotics and biomedical devices [91].

The versatility of SMPs has led to the development of advanced medical devices and dynamic tissue scaffolds. Kuang et al. developed a novel ink containing urethane diacrylate and a semicrystalline thermoplastic polymer for UV-light-assisted direct-ink-write printing. This process created a semi-interpenetrating polymer network elastomer that exhibited high-strain and self-healing capabilities. The study focused on optimizing the ink composition and UV curing parameters to achieve the desired material properties. The resulting elastomer demonstrated high stretchability (up to 600% strain), excellent shape-memory properties, and self-healing capabilities [89]. These properties are critical for applications in soft robotics, flexible electronics, and biomedical devices, where material resilience and functionality are essential. Wu and Hsu’s work on self-healing hydrogels with shape-memory properties emphasizes the applicability of 4D printing in developing dynamic, multifunctional biomedical implants [114]. The development of biocompatible stents for left atrial appendage occlusion exemplifies the potential of 4D printing in creating advanced medical devices [115]. Similarly, shape-memory vascular stents based on β-β-cyclodextrin-g-polycaprolactone, with high tensile strength and biocompatibility, exemplify the potential for advanced vascular therapies [113]. Additionally, the precise control over mechanical properties in SMPs further underscores the importance of understanding and optimizing these correlations [83]. A study focused on developing a dually crosslinked hydrogel that can achieve high stretchability, self-healing ability, and 4D printability for applications in stretchable electronics. The hydrogel demonstrated superior mechanical properties, including a fracture strain of up to 1700% and a self-healing efficiency of 88%.

#### 3.3.2. Hydrogels, Smart Inks, and Biodegradable Scaffolds

**Figure 6:**
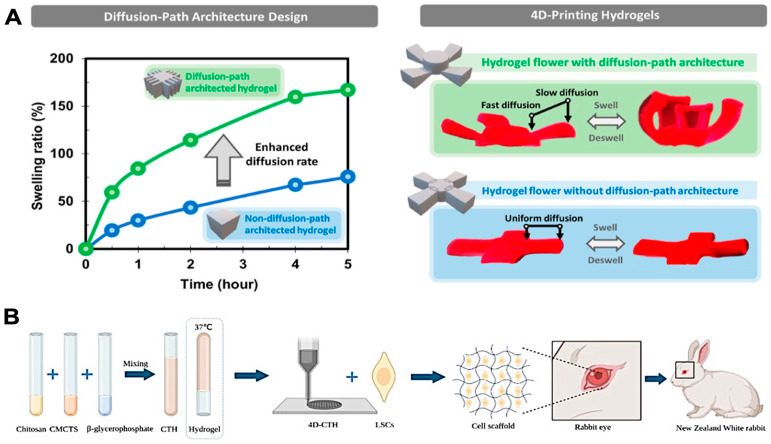
Innovative 4D bioprinting of hydrogel-based constructs for tissue engineering and regenerative medicine. (**A**) Illustration of the diffusion-path architecture in hydrogels, showing how the extended paths enhance swelling kinetics and uniformity, enabling programmable shape transformations. (**B**) Development of a 4D-printed chitosan-based scaffold carrying limbal stem cells (LSCs) to treat corneal injuries in diabetic rabbits. The scaffold promotes faster wound healing and nerve regeneration, showcasing the potential of 4D bioprinting in regenerative medicine. Reprinted from [116,118] under the terms of the Creative Commons License CC BY 4.0—https://creativecommons.org/licenses/by/4.0/ (accessed on 9 September 2024).

Hydrogels and composite materials play an equally critical role in 4D printing, particularly in biomedical applications where their stimuli-responsive behavior can be harnessed for creating adaptive structures. Pruksawan et al. developed hydrogel actuators using diffusion-path architecture design (Figure 6A) to dynamically respond to environmental stimuli [116]. This approach manipulated the swelling kinetics of hydrogels by engineering their diffusion-path architecture. By adjusting the diffusion-path length within the hydrogel, significant changes in swelling kinetics were achieved, resulting in precise control over the diffusion and transport processes within the hydrogel [116]. The outcomes included uniform properties throughout the hydrogel and programmable and reversible shape transformations, suitable for advanced 4D-printed biomedical devices and soft robotics applications. Ding et al. developed a new cell-laden bioink for 4D bioprinting, termed jammed micro-flake hydrogel (MFH). This bioink features shear-thinning, shear-yielding, and rapid self-healing properties, allowing smooth extrusion and stable constructs with high cell viability and complex geometries [117]. Their research highlights the potential for innovative 4D bioprinting applications in tissue engineering and regenerative medicine.

Similarly, Wang et al. demonstrated the use of a 4D-printed chitosan-based scaffold carrying limbal stem cells (LSCs) to treat corneal epithelium injuries in diabetic rabbits (Figure 6B). This innovative scaffold, created from a blend of chitosan and carboxymethyl chitosan (CTH) as a thermosensitive hydrogel, was printed using 4D bioprinting technology to form a porous structure with uniform pore diameters, specifically designed to improve LSC loading efficiency and local adhesion at the site of injury. The diabetic rabbits, induced by alloxan injections, exhibited delayed wound healing due to the effects of diabetes on corneal epithelial regeneration. When the LSC-loaded scaffold was applied to the surface of the injured corneas, it significantly accelerated wound healing compared to control groups, which received either LSCs without the scaffold or no treatment. Additionally, the 4D-CTH scaffold promoted corneal nerve regeneration and reduced inflammation, as evidenced by histological and immunofluorescence analyses [118]. Others developed a muscle tissue model using an electric-field-assisted 3D/4D bioprinting process with GelMA-based cell-aligned bioink, providing significant insights into tissue engineering applications [119]. These findings highlight the potential of 4D-printed scaffolds for not only corneal repair but also broader applications in tissue engineering and regenerative medicine.

Keshavarz et al. developed smart alginate inks for 3D and 4D bioprinting that showcase their potential in tissue engineering applications. By incorporating nanomaterials and chemical modifications, they created alginate bioinks with enhanced printability, bioactivity, and mechanical properties [120], leading to improved printability and bioactivity. Díaz-Payno et al. focused on a novel 4D bioprinting approach for cartilage tissue engineering using human mesenchymal stromal cells (hMSCs). The method involved creating a bilayer construct with differential swelling properties using tyramine-functionalized hyaluronan (HAT) and a composite of alginate and HAT (AHAT). This differential swelling results in shape transformation, enabling the fabrication of complex, curved structures. The study demonstrates the viability and chondrogenic potential of the bioprinted constructs, which maintained curvature and supported cartilage-like matrix production over 28 days in culture [121].

#### 3.3.3. Thermoresponsive Materials and Nano–Micro Assemblies

Thermoresponsive and magnetic-field-responsive materials have gained traction in 4D printing for their ability to create smart, adaptive structures. For example, the effects of printing temperature on poly(N-isopropylacrylamide) (pNIPAM) hydrogels further underscore the significance of optimizing printing parameters for biomedical applications [122]. A recent study highlighted the importance of using comprehensive material–process–property correlations in developing N-vinyl caprolactam (NVCL)-based hydrogels for 4D printing, focusing on the phase transitions and thermal behaviors critical for smart and responsive systems [123]. The study explored the effect of varying NVCL concentrations and crosslinking densities on the thermal responsiveness and mechanical properties of the hydrogels. Similarly, Halligan et al. investigated the modulation of the lower critical solution temperature (LCST) of poly(N-vinylcaprolactam) (PNVCL) by incorporating hydrophilic N-vinylpyrrolidone (NVP) and hydrophobic vinyl acetate (VAc) monomers, achieving temperature adjustments suitable for 4D printing applications. The study found that NVP increased the LCST above physiological temperatures, while VAc lowered it to approximately 21 °C, enabling room-temperature transformations [124]. This modulation allowed for tailored temperature-sensitive behavior in biomedical and drug delivery applications.

Lee et al. developed light-triggered in-situ gelation of hydrogels using 2D molybdenum disulfide (MoS_2_) nano assemblies [125]. The study demonstrated that NIR-triggered gelation results in highly responsive hydrogel structures with improved mechanical properties, and potential applications in phototherapy, 3D/4D printing, and therapeutic delivery, showcasing the integration of material science and process engineering in achieving desired 4D outcomes [125]. Strutynski et al. manufactured shape-memory optical fibers from thermally stretched, additively manufactured preforms [126]. The study used standard commercially available thermoplastics to produce long, continuously structured microfilaments with shape-memory abilities. The fibers were programmed to switch from temporary configurations back to user-defined shapes while maintaining efficient light transmission through multiple temperature-triggered bending/straightening cycles [126].

The latest advancements in nanotechnology, focusing on the synthesis, characterization, and practical uses of nanomaterials in 4D printing, are crucial for enhancing material properties and expanding application possibilities [127]. A group designed a magnetization drive coil based on the Helmholtz principle to achieve the magnetization of hydrogel mixed with NdFeB powder [128]. Key variables included the coil design parameters, the hydrogel composition, and the importance of accurate simulations and experimental validation in achieving uniform and dense magnetic induction distribution, essential for reliable performance in practical applications. Similarly, Sotoudeh et al. investigated the reversible performance of shape-memory composites using magnetic field stimulation [129]. The study focused on polylactic acid (PLA) combined with strontium ferrite (SrFe_12_O_19_) to create composite filaments. Finite element analysis (FEA) was used to develop a model for controlling deformation during the shape recovery process. Key parameters included printing speed, layer height, and the layer infill number at 90°. The outcomes demonstrated high accuracy in shape recovery, emphasizing the importance of combining computational models with experimental approaches. To illustrate the differences and applications of various materials, Table 2 presents a summary of materials used in 3D and 4D printing.

#### 3.3.4. Modeling, Simulation, and Optimization

Modeling and simulation techniques are essential for optimizing the behavior of 4D-printed materials. Zhao et al. developed comprehensive models to predict the shape-memory performance and decay behaviors of SLA-printed parts [134]. By employing FEA, they simulated the shape-memory behavior and predicted performance over time based on material composition and printing parameters. Their study highlighted the importance of accurate modeling for designing materials with predictable and reliable responses to external stimuli. Similarly, Zheng et al. combined experimental results with FEA to predict the performance of soft microactuators [102]. This approach ensured reliable and repeatable actuation under various conditions, further underscoring the necessity for accurate simulations to design materials with predictable and reliable responses to external stimuli. These studies emphasize the critical role of modeling in enhancing the practical applications of 4D-printed structures.

In addition to simulation, process optimization is essential for achieving uniform material properties. Tan et al. introduced a power compensation strategy to create homogeneous microstructures in poly(N-isopropylacrylamide) (PNIPAM) hydrogels [135]. By employing a piecewise linear approximation and stepwise power adjustments, the team calibrated the system using deswelling ratios and ensured consistent polymerization across varying heights. Experimental validation confirmed the effectiveness of this strategy, with saturation of crosslinking density observed at powers above 45% [135], and this strategy successfully produced 4D-printed hydrogel structures with uniform crosslinking density, confirmed through experimental validation. Advancements in programming techniques also play a key role in expanding the capabilities of 4D printing. For example, Yue et al. introduced cold-programmed shape-morphing structures using grayscale digital light processing to simplify the programming process and broaden the range of achievable configurations [136]. This approach offers greater efficiency and versatility in 4D printing, enabling more complex and adaptable structures.

Furthermore, the incorporation of dynamic, semi-orthogonal chemical modifications in microgel scaffolds, as presented by Miksch et al., allowed for tunable void fractions and programmable material properties, essential for responsive and adaptive 4D structures [133]. The authors optimized 4D-printed microgel scaffolds using modular chemistries like thiolene for spatial and temporal control, photodegradable crosslinks for tuning scaffold porosity, and high-throughput encapsulation techniques for combined 2.5D and 3D cell cultures. Orthogonal degradation cues were introduced, allowing stimuli-responsive delivery of proteins and small molecules to precisely control the scaffold’s mechanical and biochemical properties [133].

#### 3.3.5. Applications in Other Engineering Fields

Engineering fields other than biomedical engineering also benefit from 4D printing through adaptive structures and components. Wang et al. demonstrated direct 4D printing of ceramics driven by hydrogel dehydration, producing high-strength components capable of withstanding extreme conditions [137]. These characteristics make 4D-printed ceramics highly suitable for applications in aerospace and automotive industries, where materials must endure extreme environments and stresses. The adaptability of these components is crucial for improving the resilience and performance of engineering systems in these demanding sectors.

Similarly, the use of SMPs has expanded the possibilities for adaptive structures in engineering. These materials enable the development of components that can respond to environmental changes, such as temperature or pressure variations, which is highly valuable for aerospace and automotive systems [82]. For example, Weng et al. explored the potential of glass-fiber-regulated shape-shifting structures, which offer high stiffness and durability, making them ideal for applications requiring both mechanical strength and adaptability [106].

The versatility of 4D printing extends to other areas as well. Research into pNIPAM hydrogels, which exhibit significant swelling and deswelling behaviors, suggests potential applications in smart clothing and accessories that can adjust their properties based on environmental conditions [122]. This suggests potential for 4D printing in the fashion and consumer goods industries, where adaptability to environmental stimuli could enhance user comfort and functionality. Additionally, the development of multi-color 4D-printed SMPs enables the creation of dynamic, aesthetically pleasing consumer products [138]. The incorporation of shape-memory effects in everyday items highlights the versatility and practicality of 4D printing in enhancing consumer experiences.

In the field of robotics, Makki et al. demonstrated the use of virgin polymers in 4D printing for creating dynamic robotic components [139]. Recent advancements in 4D-printed liquid crystal elastomers with controllable orientation gradients have allowed for complex deformation modes, enhancing the adaptability of robotic systems [140]. This ability to fine-tune the behavior of materials during deformation makes 4D printing particularly valuable in robotics, where responsive materials are crucial for developing more versatile, adaptable machines.

Finally, the development of chitosan and whey protein bioinks for 3D and 4D printing has shown significant potential for applications in the food industry [141]. The combination of chitosan and whey protein offers excellent biodegradability and biocompatibility, making them suitable not only for creating responsive and functional food products but also for developing advanced biomaterials in tissue engineering and biomedical research.

### 3.4. Prospects and Challenges in 4D Printing

While 4D printing holds great promise, several technical challenges must be addressed before its full potential can be realized. One significant limitation is the development of smart materials with consistent and reliable performance. Many current smart materials, such as SMPs and hydrogels, exhibit variability in their response to stimuli, which can affect the reliability and repeatability of 4D-printed structures [18,114]. This variability creates difficulties in ensuring predictable behavior under different conditions, making it challenging to produce structures that perform uniformly over time and across applications. Additionally, the integration of multiple smart materials into a single 4D-printed structure presents challenges in terms of material compatibility and process optimization. For example, combining materials with different thermal or mechanical properties can lead to issues such as delamination or uneven shape transformation [142].

Another key challenge lies in achieving precise control over printing parameters to ensure the desired material properties and shape transformations. Variability in factors such as printing temperature, speed, and layer thickness can significantly impact the performance of 4D-printed parts [116]. Moreover, the scalability of 4D printing processes for industrial applications remains a critical hurdle. Ensuring that large-scale 4D-printed structures maintain the same level of precision and functionality as smaller prototypes is a complex task that requires further research and development. Another major challenge is the durability and longevity of 4D-printed materials. While some smart materials exhibit excellent initial performance, their long-term stability under repeated cycles of stimuli exposure remains uncertain. This is particularly important for applications in biomedical and aerospace fields, where materials must withstand harsh environments and prolonged use [4,97].

Despite these challenges, the field of 4D printing is bright, driven by ongoing research and innovation. One promising area of advancement is in material–process–property correlations, where a better understanding of the interplay between material properties and printing processes can lead to more predictable and controllable 4D printing outcomes. This involves the use of advanced modeling and simulation techniques to optimize the design and fabrication of 4D-printed structures [117,133]. Additionally, innovations in printing technology, such as multi-material printing and high-resolution additive manufacturing, are expanding the capabilities of 4D printing by enabling the creation of more complex and functional structures [142].

The integration of artificial intelligence (AI) and machine learning (ML) into 4D printing is another exciting frontier. AI and ML could be used to optimize printing processes, predict material behaviors, and even design complex structures with minimal human intervention, reducing the trial-and-error approach that is currently prevalent in the field. This integration could lead to more efficient and accurate 4D printing, ultimately improving the technology’s scalability and reliability.

In the biomedical field, research is focused on developing new applications for 4D printing, such as personalized medical implants and responsive drug delivery systems. The ability to create biocompatible and biodegradable materials that can change shape or release drugs in response to physiological conditions holds great promise for improving patient outcomes [18,114].

In summary, while 4D printing faces several technical and material challenges, ongoing research and innovation are paving the way for significant advancements. Advancements in printing technology, the development of new smart materials, and the improved understanding of material–process–property correlations are all contributing to the prospects of 4D printing. As these innovations continue to unfold, 4D printing is expected to revolutionize various industries, from biomedical engineering to aerospace, consumer products, and robotics. This could lead to significant advancements in patient-specific therapies and more effective treatment options.

Simply put, while 4D printing faces several technical and material challenges, ongoing research and innovation are paving the way for significant advancements. The development of new smart materials, improvements in printing technology, and a deeper understanding of material–process–property correlations are all contributing to the future of 4D printing. As these innovations continue to emerge, 4D printing is expected to revolutionize industries such as biomedical engineering, aerospace, consumer products, and robotics, transforming the way adaptive and functional structures are designed and manufactured.

## 4. Summary and Discussion

The application of 4D printing in biomedical devices has revolutionized several fields, introducing the ability to create dynamic and responsive structures. Unlike traditional 3D printing, which produces static objects, 4D printing enables the fabrication of medical devices that can change shape or properties in response to external stimuli such as temperature, pH, or moisture. This capability is particularly advantageous for creating patient-specific medical devices, such as self-expanding stents, shape-memory sutures, and drug delivery systems that release medication in a controlled manner. Furthermore, 4D printing utilizes smart materials like shape-memory polymers and hydrogels, which respond actively to environmental conditions, enhancing the range of possible biomedical applications. These materials also ensure that 4D-printed devices can interact safely with human tissues without causing adverse reactions, making the technology especially valuable for biomedical engineering.

While 3D printing primarily focuses on creating static objects layer by layer, utilizing techniques like Fused Deposition Modeling and Stereolithography, 4D printing incorporates the dimension of time, allowing structures to undergo transformation post-production. The key difference lies in the adaptive nature of 4D printing, which enables it to create multifunctional, stimuli-responsive devices tailored to patient-specific needs. In addition, the integration of smart materials allows 4D-printed objects to dynamically respond to changes in the body, a feature not present in traditional 3D printing. As such, while 3D printing remains a powerful tool for prototyping, manufacturing, and producing static medical devices, 4D printing holds particular promise for areas that require adaptability, such as soft robotics, self-healing materials, and dynamic implants.

The use of biodegradable polymers in 4D printing further addresses a growing concern regarding environmental sustainability. Polymers such as polylactic acid, polycaprolactone, and polyhydroxyalkanoates offer excellent biocompatibility and biodegradability, allowing medical devices, like tissue engineering scaffolds and drug delivery systems, to degrade naturally over time. This feature reduces the need for device retrieval surgeries and diminishes environmental impact. Such advancements are particularly significant in both medical and environmental contexts, positioning 4D printing as a sustainable option for developing patient-specific, eco-friendly solutions.

However, despite these significant strides, there are still critical challenges in realizing the full potential of 4D printing. One of the primary technical hurdles is the development of smart materials that exhibit reliable, consistent performance. For example, both SMPs and hydrogels can show variability in their responses to stimuli, which affects the reliability of the printed objects in medical settings. Additionally, integrating different smart materials into a single 4D-printed structure raises concerns about material compatibility, especially when combining components with varying thermal and mechanical properties. This often leads to issues like delamination or uneven shape transformation. Furthermore, optimizing the printing process to ensure precision and consistency in large-scale applications remains a significant obstacle. While laboratory-scale prototypes of 4D-printed devices show great promise, scaling these techniques for industrial use while maintaining accuracy and reliability will require further advancements.

While both 3D and 4D printing offer immense potential for biomedical applications, one of the main challenges lies in ensuring the safety and biocompatibility of the materials used. Biocompatibility is crucial because materials must interact safely with biological tissues without eliciting harmful responses. This review highlights significant progress in using biocompatible polymers like PLA and PEEK, but more attention should be given to the challenges of long-term safety. For example, issues like the potential for degradation byproducts, inflammatory responses, and ensuring that bioresorbable materials break down into non-toxic components need further exploration. Ensuring that newly developed medical devices meet rigorous safety standards is critical for widespread clinical adoption. Therefore, more research and regulatory oversight are required to address these concerns and ensure that the materials used are both effective and safe for patients in the long term.

Another key area requiring attention is the long-term durability of 4D-printed materials. Many smart materials exhibit excellent initial performance but can degrade when subjected to repeated cycles of external stimuli over time. For applications in biomedical and aerospace industries, where devices must endure harsh environments, improving the longevity and reliability of these materials is essential.

In summary, while 4D printing faces several technical and material challenges, ongoing research is leading to significant breakthroughs. Advancements in material–process–property correlations, the development of new smart materials, and the continuous improvement of 4D printing technologies all contribute to the rapid growth of this field. As the field progresses, 4D printing is expected to revolutionize industries beyond biomedicine, extending its impact to fields such as aerospace, consumer products, and robotics. The integration of artificial intelligence (AI) and machine learning (ML) into 4D printing processes is an exciting trend, offering new possibilities for optimizing printing workflows, predicting material behavior, and automating the design of complex adaptive structures. With these ongoing innovations, 4D printing is set to transform the future of manufacturing, ushering in a new era of smart, dynamic, and sustainable technologies.

## 5. Future Research Directions

Advancing the field of 3D and 4D printing will depend on several pivotal areas of research and development. One of the primary focuses will be the creation of next-generation smart materials with enhanced responsiveness and longevity. These materials need to withstand diverse environmental conditions while maintaining their functional integrity over extended periods. Innovations in polymer chemistry and composite materials are expected to drive this progress, offering solutions that meet the stringent demands of various applications, particularly in the biomedical sector.

In the realm of personalized medicine, 4D printing holds transformative potential. Future research will likely delve into developing highly sophisticated 4D-printed medical devices tailored to individual patient needs. These devices could dynamically respond to physiological changes, such as healing processes, thereby optimizing their therapeutic efficacy. The integration of bioprinting technologies to fabricate functional tissues and organs represents another frontier, potentially revolutionizing regenerative medicine and tissue engineering by providing custom-made, biocompatible solutions for organ replacement and repair.

Sustainability will continue to be a critical consideration, driving efforts to develop eco-friendly materials and manufacturing processes. This includes not only biodegradable and biocompatible polymers but also innovative approaches to reduce the environmental footprint of the printing processes themselves. Enhancing the energy efficiency of 3D and 4D printing and minimizing material waste are essential goals for future research, contributing to a more sustainable manufacturing paradigm.

Interdisciplinary collaboration will play a crucial role in overcoming the complex challenges associated with these advanced manufacturing technologies. Bringing together expertise from materials science, engineering, biology, and computational sciences can foster innovative solutions that leverage the strengths of each discipline. This collaborative approach is essential for tackling the multifaceted problems in developing and implementing cutting-edge 3D and 4D printing technologies.

The integration of artificial intelligence (AI) and machine learning (ML) into 3D and 4D printing processes stands out as a promising avenue. These technologies can optimize printing parameters, predict material behavior, and design complex structures with unprecedented precision and efficiency. AI- and ML-driven approaches could significantly reduce trial and error in material development and process optimization, accelerating innovation and improving the reliability of printed products.

The future of 3D and 4D printing is poised for significant advancements through continuous material innovation, the adoption of AI and ML technologies, and a steadfast commitment to sustainability and personalized healthcare. These developments will expand the scope and impact of additive manufacturing, driving progress across various industries and setting new standards for technological innovation and environmental responsibility.

## Figures and Tables

**Table 1:** Summary of various 3D printing techniques in biomedical applications.

3D Printing Methodology	Material	Application	Highlights and Advantages	Limitations	Resolution (μm) and Printing Speed (mm/h)	Approximate Cost (Materials/Equipment)	References
Fused Deposition Modeling (FDM)	Thermoplastic filaments	Bone scaffolds, prosthetics, drug delivery	Improved resolution and surface finish. Cost-effective, multi-material capabilities, low cost	Lower resolution, anisotropic mechanical properties. Limited to thermoplastics	100–300, 150–300	Materials: 50–150 USD USD/kg; Equipment: USD 500–USD 20,000	[24,26,27]
Stereolithography (SLA)	Photopolymer resins	Surgical models, dental prosthetics, scaffolds	High resolution and smooth surface finish. Detailed anatomical representations	Brittle parts, limited material choices, post-processing required	25–100, 150–300	Materials: 100–200 USD/kg; Equipment: USD 2000–USD 50,000	[26,29,59]
Selective Laser Sintering (SLS)	Metal and polymer powders	Aerospace, automotive, medical implants	Produces strong, durable parts. Little support is needed for polymer materials	High cost, requires post-processing for metal heat management	100–200, 10–50	Materials: 100–400 USD/kg; Equipment: USD 50,000–USD 1,000,000+	[26,34]
Digital Light Processing (DLP)	Photopolymer resins	Microfluidic devices, tissue scaffolds	High precision, fast curing. Ideal for complex microfluidic devices	Limited to photopolymer resins, brittle parts	25–50, 200–500	Materials: 100–200 USD/kg; Equipment: USD 3000–USD 50,000	[35,60]
PolyJet	Photopolymers (rigid and flexible resins)	Surgical models, dental prosthetics, anatomical models, prosthetics	High resolution, multi-material capabilities, smooth surface finish, color and texture variation	Limited mechanical strength, high material costs	16–85, 100–400	Materials: 150–400 USD/kg; Equipment: USD 20,000–USD 100,000	[39,40,41]
Inkjet Bioprinting	Bioinks, cells	Tissue engineering, drug delivery	High precision in cell placement. Non-contact technique, high precision	Potential nozzle clogging, slower printing speed. High cost	50–100, 15–20	Materials: 200–500 USD/mL; Equipment: USD 50,000–USD 200,000	[24,25,26]
Laser-Induced Forward Transfer (LIFT)	Cells, various materials	Cellular constructs	Precise cell placement, high resolution. Avoids nozzle clogging	Requires laser setup, limited throughput. High cost	<10, 10–100	Materials: Variable; Equipment: USD 100,000–USD 1,000,000+	[34]
Direct Ink Writing (DIW)	Ceramics, hydrogels	Bone scaffolds, drug delivery	High structural integrity. Controlled deposition, low cost	Limited resolution compared to other methods	100–500, 10–50	Materials: 100–200 USD/kg; Equipment: USD 5000–USD 50,000	[24,25,26]

**Table 2:** Summary of materials used in 3D and 4D printing.

Material	Properties	Applications	Advantages	Disadvantages	Reference
Polymers	Versatile, processable, can be tailored for mechanical, chemical, and biological properties	Biomedical devices, flexible electronics, smart materials	Customizable, cost-effective, suitable for a wide range of applications	May have limited biodegradability, some polymers require high processing temperatures	[1,3,9,24]
Hydrogels	Highly absorbent, biocompatible, responsive to environmental stimuli (e.g., pH, temperature)	Tissue engineering, drug delivery, minimally invasive implants	Biocompatible, can be designed for specific responses, suitable for various medical applications	May degrade over time, mechanical properties can be limited, sensitive to environment	[12,18,97,98,104]
Composites	Enhanced mechanical and functional properties, often incorporate fibers or other materials	Bone scaffolds, drug delivery systems, high-performance components	Improved mechanical properties, multifunctional, suitable for demanding applications	Complex manufacturing processes, potential issues with material compatibility	[24,55,59,130,131]
Shape-Memory Polymers (SMPs)	Ability to return to pre-defined shape upon exposure to stimuli (e.g., heat, light, magnetic fields)	Dynamic and responsive structures, personalized medical devices	Programmable transformations, biocompatible, can create complex shapes	Potential variability in response, long-term stability under repeated cycles is uncertain	[5,11,18,82,88]
Bioinks	Mixtures of cells and biomaterials, high precision in cell placement, support cell viability	Regenerative medicine, personalized drug delivery, tissue-like structures	Support cell growth, high precision, can create complex and functional structures	Sensitive to processing conditions, potential for cell viability issues during printing	[24,25,117,132,133]

## Data Availability

No new data were created or analyzed in this study. Data sharing is not applicable to this article.
